# Automated Detection of Dysplasia: Data Mining from Our Hematology Analyzers

**DOI:** 10.3390/diagnostics12071556

**Published:** 2022-06-26

**Authors:** Jaja Zhu, Sylvain Clauser, Nicolas Freynet, Valérie Bardet

**Affiliations:** 1 Service d’Hématologie-Immunologie-Transfusion, CHU Ambroise Paré, APHP.Paris Saclay, Université Versailles Saint Quentin-Université Paris Saclay, 92100 Boulogne-Billancourt, France; jaja.zhu@aphp.fr (J.Z.); sylvain.clauser@aphp.fr (S.C.); 2 Département d’Hématologie et Immunologie Biologiques, GHU Henri Mondor, Université Paris-Créteil, 94000 Créteil, France; nicolas.freynet@aphp.fr

**Keywords:** myelodysplastic syndrome, hematology analyzer, cell population data, complete blood count, leukocyte differential

## Abstract

Myelodysplastic syndromes (MDSs) are clonal hematopoietic diseases of the elderly, characterized by chronic cytopenia, ineffective and dysplastic hematopoiesis, recurrent genetic abnormalities and increased risk of progression to acute myeloid leukemia. Diagnosis on a complete blood count (CBC) can be challenging due to numerous other non-neoplastic causes of cytopenias. New generations of hematology analyzers provide cell population data (CPD) that can be exploited to reliably detect MDSs from a routine CBC. In this review, we first describe the different technologies used to obtain CPD. We then give an overview of the currently available data regarding the performance of CPD for each lineage in the diagnostic workup of MDSs. Adequate exploitation of CPD can yield very strong diagnostic performances allowing for faster diagnosis and reduction of time-consuming slide reviews in the hematology laboratory.

## 1. Introduction

MDSs are clonal hematopoietic diseases of the elderly, characterized by chronic cytopenia, ineffective and dysplastic hematopoiesis, recurrent genetic abnormalities and increased risk of progression to acute myeloid leukemia. MDSs are among the most diagnostically challenging of myeloid neoplasms, especially in terms of their distinction from the numerous other non-neoplastic causes of cytopenia. The updated *World Health Organization (WHO) Classification of Tumours of Haematopoietic and Lymphoid Tissues* [1] still considers the thresholds for cytopenias established in the original International Prognostic Scoring System (IPSS) [2] for risk stratification (hemoglobin concentration <10 g/dL, platelet count <100 × 10^9^/L and absolute neutrophil count (ANC) <1.8 × 10^9^/L). However, an MDS diagnosis may be made in patients with a milder degree of anemia or thrombocytopenia [3]. A challenge of routine laboratory complete blood counts (CBCs) is to correctly identify MDS patients while simultaneously avoiding excess smear reviews. Considering the low frequency of MDSs [4,5], the International Society for Laboratory Hematology [6] only recommended a blood smear review if hemoglobin level <7 g/dL or mean corpuscular volume (MCV) >105 fL or absolute neutrophil count (ANC) <1 × 10^9^/L or platelet count <100 × 10^9^/L. Of course, this approach, based on medico-economic considerations, does not allow optimal screening of MDSs. To optimize slide review and enhance the sensitivity of MDS detection, cell population data parameters (CPD) have been explored for years and have demonstrated their added value in the laboratory diagnostic work-up of MDSs [7]. The latest generations of hematology analyzers provide new diagnostic tools that have an increased impact in the screening of MDSs from a CBC. A brief overview of the current available techniques is given below before discussing their contribution to the MDS work-up from the hematology laboratory’s point of view. 

## 2. Cell Population Data

**Figure 1:**
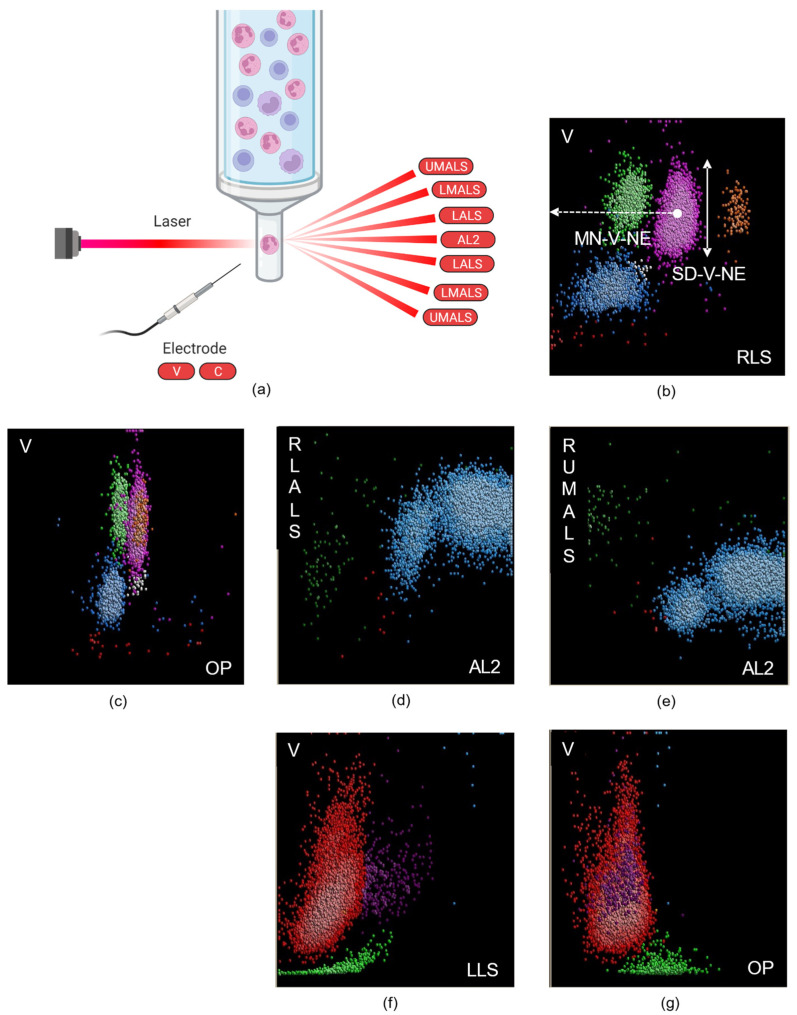
Illustration of the Coulter technology. (**a**) The Coulter UniCel DxH 800 analyzer measures cell volume (V) through the V impedance and the internal complexity or opacity (OP) of the cell with the C radio frequency. The light scattering is measured at different angles: upper-median-angle light scatter (UMALS), lower-median-angle light scatter (LMALS), low-angle light scatter (LALS) and axial-loss light scatter (AL2). The fifth light scatter channel, median-angle light scatter (MALS), is the sum of the UMALS and LMALS regions. For each parameter, mean (MN) and standard deviation (SD) are collected as cell population data (CPD). Created with BioRender.com. (**b**) Leukocyte differential: scatterplot of volume (V) versus rotated light scatter (RLS) identifies five subpopulations: neutrophils (NEs) in pink, eosinophils in orange, basophils in white, lymphocytes in blue and monocytes in green. CPD from volume are illustrated: MN-V-NE (dotted arrow) represents the mean channel value of NE population volume, SD-V-NE (vertical arrow) represents the standard deviation of volume for this population. (**c**) Leukocyte differential: scatterplot of volume (V) versus opacity (OP). Neutrophils, eosinophils and basophils have a similar opacity but basophils have a decreased volume. (**d**) Nucleated red blood cell (NRBC) plot: scatterplot of rotated low-angle light scatter (RLALS) versus axial-loss light scatter (AL2) showing in blue the leukocytes, in green macroplatelets and platelet clumps (and debris) and a few sparse red events corresponding to NRBCs. (**e**) Nucleated red blood cell (NRBC) plot: scatterplot of reflected upper-median-angle light scatter (RUMALS) versus AL2 showing in blue the leukocytes, in green the platelets (macroplatelets, platelet clumps) and a few sparse red events corresponding to NRBCs. (**f**) Scatterplot of volume (V) versus linear light scattering (LLS) showing in green platelets (and debris), in purple reticulocytes and in red mature RBCs. (**g**) Scatterplot of volume (V) versus opacity (OP) showing in green platelets (and debris), in purple reticulocytes and in red mature RBCs.

Automated hematology analyzers (HAs) generate both quantitative and qualitative data, using various techniques for CBC and white blood cell (WBC) differential counting. The Coulter principle, patented by Wallace Coulter in 1953, provided the basis for the first automated method of cell counting. Beckman Coulter HAs perform impedance-based CBC and use a differential based on a combination of three physical parameters: flow cell volume (V), conductivity (C) and light scatter measurements (S) obtained by the VCS flow technology. Hemoglobin (Hb) is determined using spectrophotometry. Cell counts, hemoglobin content and cellular indices, such as MCV or mean platelet volume (MPV), can also be obtained from optical methods. The scattered light is directly related to the size (area), surface irregularities and refractive index of the illuminated particle or cell. At a low angle of scattered light detection, the area of the cell represents the highest input source to extrapolate cell volume. At high-angle detection of scattered light, the refractive index is the major input, therefore reflecting the granularity of the particle or cell. Based on this principle, the low-angle scattered-light measurements provide better discrimination of particles that have the same volume but different contents, i.e., large platelets are discriminated from small red blood cells (RBCs) such as schistocytes [8]. In addition to volume and conductivity measurements, the Unicel DxH 800 from Beckman Coulter (Beckman-Coulter, Brea, CA) generates an additional five angles of laser scatter, including median-angle light scatter (MALS), upper-median-angle light scatter (UMALS), lower-median-angle light scatter (LMALS), low-angle light scatter (LALS) and axial-loss light scatter (AL2) as illustrated in Figure 1a. MALS, LMALS and UMALS provide information regarding the granularity and membrane surface, whereas AL2 measures cellular transparency and LALS cellular complexity. CPD derive from volume and conductivity measurements and from the new light scatter parameters (Figure 1b–g). Seven different CPD are thus available from WBCs (neutrophils, eosinophils, lymphocytes, monocytes), early granulated cells (EGCs), reticulocytes and nucleated red blood cells (NRBCs). 

**Figure 2:**
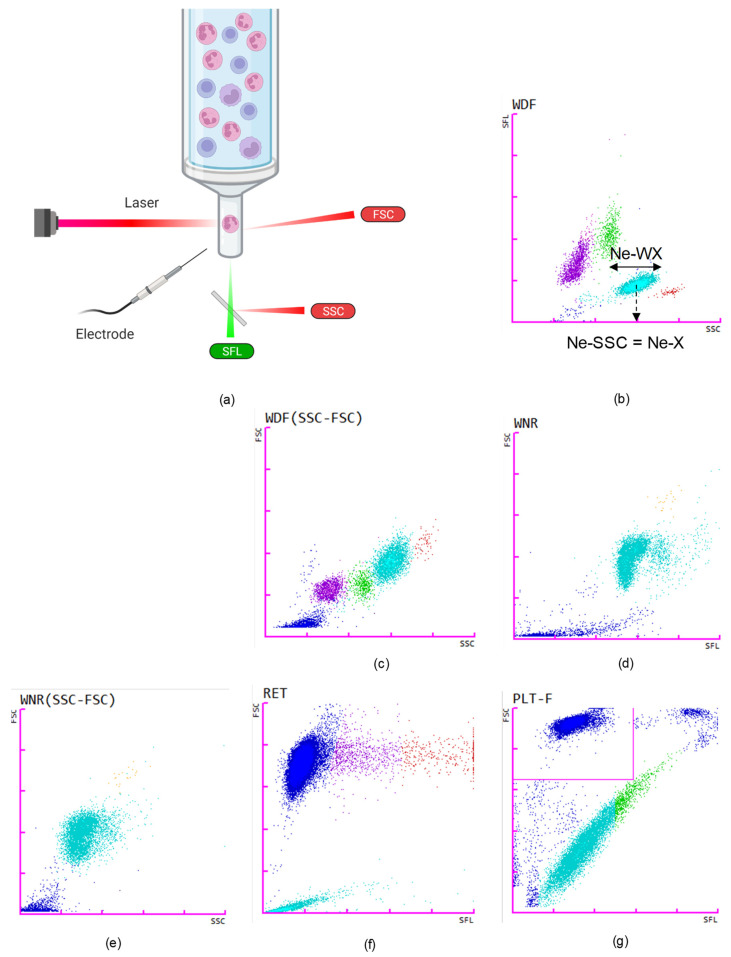
Illustration of the Sysmex technology. (**a**) The Sysmex-XN applies fluorescence-flow cytometry on cells to collect information on internal complexity (side scatter, SSC), nucleic acid content (fluorescence, SFL) and cell size (forward scatter, FSC. Created with BioRender.com. (**b**) Position of different cell populations on the white blood cell differential scatterplot (WDF). X-axis shows side scatter (SSC) of laser light, Y-axis represents side fluorescence (SFL). The different types of leukocytes are represented: light blue for neutrophils, pink for lymphocytes, green for monocytes, red for eosinophils and dark blue for platelets and debris. Neutrophil scattering parameters are illustrated by the dotted arrow for granularity (SSC) on the X-axis (Ne-X) and the horizontal arrow for neutrophil side scatter area distribution width (Ne-WX). (**c**) White blood cell differential scatterplot (WDF). X-axis shows side scatter (SSC) of laser light, Y-axis represents forward scatter (FSC). (**d**) White count and nucleated red blood cell scatterplot (WNR) showing in light blue lysed leukocytes, in yellow basophils. If present, nucleated red blood cells (NRBCs) are identified in purple. X-axis shows side scatter (SSC) of laser light, Y-axis represents side fluorescence (SFL). (**e**) White count and nucleated red blood cell scatterplot (WNR) showing in light blue lysed leukocytes and in yellow basophils. If present, nucleated red blood cells (NRBCs) are identified in purple. X-axis shows side scatter (SSC) of laser light, Y-axis represents forward scatter (FSC). (**f**) Reticulocytes scatterplot showing high-, medium- and low-fluorescent reticulocytes in red, orange and purple, respectively, and mature RBCs in dark blue. X-axis shows side scatter (SSC) of laser light, Y-axis represents forward scatter (FSC). (**g**) Fluorescence platelet count scatterplot showing in green immature platelets (ÏPF) and in light blue platelets. At the top of the graph, red blood cells appear in dark blue.

This optical flow cytometry technique has been combined with selective lysis and fluorescence flow cytometry in Sysmex and Abbott HAs. The XN-series (Sysmex Corporation, Kobe, Japan) technology is based on fluorescence flow cytometry using blood-cell membrane surfactant reagents and different fluorescent dyes that specifically stain nucleic acids and proteins. This fluorescence flow cytometry is used for WBC differential count and a combination of side scatter (SSC, X-axis, proportional to the internal complexity), fluorescence intensity (SFL, Y-axis, proportional to nucleic acid content) and forward scatter (FSC, Z-axis, related to cell size), for identifying the different WBC subpopulations (Figure 2a). These three measurements, along with their distribution width (W), are combined to generate the CPD for neutrophils, eosinophils, basophils, lymphocytes and monocytes as illustrated in Figure 2b–e. This technology is also applied to RBCs if a reticulocyte count is required and to the fluorescence platelet count when triggered, generating information on hemoglobin content and RNA content of reticulocytes and platelets (Figure 2f–g). “Young” platelets are, thus, identified as the immature platelet fraction (IPF%). 

**Figure 3:**
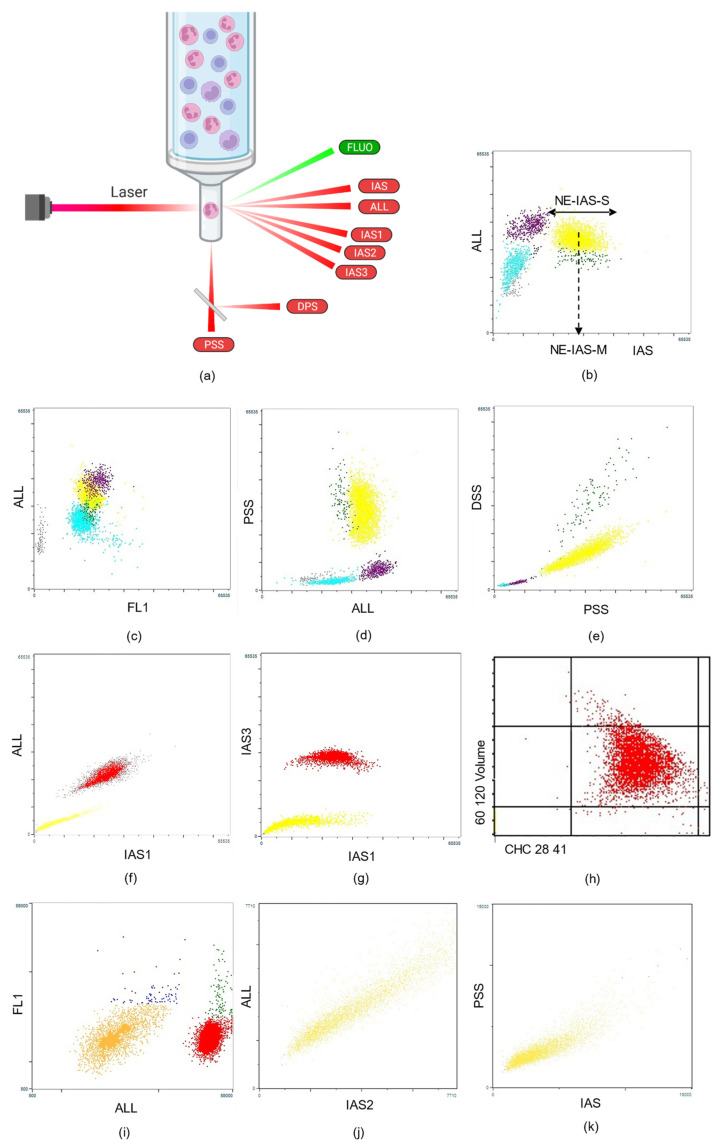
Illustration of the Abbot technology. (**a**) Alinity-hq from Abbott uses seven light scatter detectors to determine various cellular features: axial light loss (ALL) related to size, intermediate-angle scatter (IAS) related to cellular complexity, polarized side scatter (PSS) related to nuclear lobularity/segmentation and depolarized side scatter (DSS) allowing for specific identification of eosinophil granulocytes. The three narrow-angle light scatter detectors IAS1, IAS2 and IAS3 provide information on the volume, hemoglobin content and granularity of red blood cells (RBCs) and platelets. Created with BioRender.com. (**b**) Leukocyte differential on the axial light loss (ALL) versus intermediate-angle scatter (IAS) scatterplot: neutrophils are shown in yellow, lymphocytes in light blue, monocytes in purple, eosinophils in green, basophils in black and nucleated red blood cells in red, if present. Neutrophil scattering parameters are illustrated by the dotted arrow for granularity (IAS) on the X-axis (NE-IAS-M) and the horizontal arrow for neutrophil side scatter standard deviation (NE-IAS-S). (**c**) Leukocyte differential on the axial light loss (ALL) versus fluorescence (FL1) scatterplot: neutrophils appear in yellow, lymphocytes in light blue, monocytes in purple, eosinophils in green and basophils in black. Gray dots are non-DNA-containing material such as platelet clumps or lyse-resistant RBCs. (**d**) Leukocyte differential on polarized side scatter (PSS) versus axial light loss (ALL) scatterplot: neutrophils appear in yellow, lymphocytes in light blue, monocytes in purple, eosinophils in green and basophils in black. (**e**) Leukocyte differential on depolarized side scatter (DSS) versus polarized side scatter (PSS) allows for specific identification of eosinophil granulocytes in green. (**f**) RBCs (red) and platelets (yellow) on axial light loss (ALL) versus intermediate-angle scatter 1 (IAS1) scatterplot. (**g**) RBCs (red) and platelets (yellow) on intermediate-angle scatter 3 (IAS3) versus intermediate-angle scatter 1 (IAS1) scatterplot. (**h**) RBC scatterplot showing the distribution of cell hemoglobin concentration (CHC) versus volume. Microcytic RBCs (MICs) have a volume inferior to 60 fL, while that of macrocytic RBCs (MACs) is superior to 120 fL. Hypochromic RBCs (HYPs) have a CHC inferior to 28 g/dL and hyperchromic RBCs (HPRs) superior to 41 g/dL. (**i**) RBC and platelet fluorescence (FL1) versus axial light loss (ALL) scatterplot showing in green reticulocytes separated from mature RBCs in red, and reticulated platelets in blue separated from platelets in orange. (**j**) Platelets (yellow) axial light loss (ALL) versus intermediate-angle scatter 2 (IAS2) scattergram. (**k**) Platelets (yellow) polarized side scatter (PSS) versus intermediate-angle scatter (IAS) scattergram.

The Multi-Angle Polarized Scatter Separation (MAPSS™) technology from Abbott uses four light scatter detectors to determine various cellular features: 0° axial light loss (ALL) related to size, 0–10° intermediate-angle scatter (IAS), related to cellular complexity, 90° polarized side scatter (PSS), related to nuclear lobularity/segmentation, and 90° depolarized side scatter (DSS), allowing for specific identification of eosinophil granulocytes. The recent Alinity-hq (Abbott, Santa Clara, CA), through three additional narrow-angle light scatter detectors (IAS1: 2.5–4.5°, IAS2: 4.5–5.5° and IAS3: 5.5–7.5°) (Figure 3a), provides information on volume, hemoglobin content and granularity, in a cell-by-cell fashion, after isovolumetric spherization of RBCs and platelets. These detectors were also applied to WBCs, leading to the generation of CPD from the three lineages (Figure 3b–k). All these techniques have contributed to measuring and reporting a variety of innovative CPD, expressed as the mean (MN or M), standard deviation (SD), distribution width (W), distribution skewness (DS) or distribution kurtosis (DK) depending on the manufacturer. The CPD parameters can detect morphological changes in the different blood lineages and assist in the differential diagnosis of anemia [9], the early diagnosis of acute infection or sepsis [10], the detection of neutrophil dysplasia [11,12,13,14] or the differential diagnosis of peripheral thrombocytopenia [15,16]. 

### 2.1. Leukocyte-Derived CPD 

The presence of hypogranulated/degranulated neutrophils, a hallmark of dysplasia in the context of MDSs or chronic myelomonocytic leukemia (CMML) is detected through variations of different CPD. Structural neutrophil dispersion (Ne-WX) from Sysmex-XN, was shown to be increased in CMML [13] and MDS patients [11] and was included in the mono-dysplasia score, described by Schillinger et al., and the MDS-CBC score, described by Boutault et al. This score was established on a cohort of 109 newly diagnosed MDS patients and 399 cytopenic patients older than 50 years. This score incorporated three parameters: absolute neutrophil count (ANC), Ne-WX and MCV and was calculated as soon as a cytopenia was detected (hemoglobin <13 g/dL in men or <12 g/dL in women, platelet count <150 × 10^9^/L, ANC <1.8 × 10^9^/L). The MDS-CBC score demonstrated a high sensitivity of 86% and a specificity of 88% for the screening of MDSs. Of the 28 CPD derived from leukocytes or NRBCs analyzed on DxH 800, Kim et al. identified on 119 samples from 37 MDS patients and 583 samples from three non-clonal cytopenia groups, 21 CPD that were significantly different between MDS patients and the three non-clonal cytopenia groups [17]. Twelve of them (SD-V-NE, MN-MALS-NE, SD-MALS-NE, MN-UMALS-NE, SD-UMALS-NE, MN-LMALS-NE, SD-AL2-NE, MN-V-EO, SD-V-EO, MN-UMALS-EGC, MN-LALS-NNRBC and MN-AL2-NNRBC), plus RDW, were included in a diagnostic score. This multiparametric scoring of MDS provides a high diagnostic power among cytopenia samples for diagnosing MDS with an overall sensitivity (Se) of 92.4% and a specificity (Spe) of 85.4%. Nevertheless, on DxH 800, Shestakova et al. studied seven VCS parameters in neutrophils, eosinophils, lymphocytes, monocytes and EGC on a cohort of 43 MDS and 21 control individuals [14]. The best predictor of MDS was SD-UMALS-NE (Se 77% and Spe 82% alone), but the other six SDs revealed a good accuracy in predicting MDS (SD-V-NE, SD-LMALS-NE, SD-AL2-NE, SD-MALS-MO, SD-UMALS-MO, and SD-MALS-EO). Recently, Ravalet et al. [12] confirmed, using a DxH 800 on a cohort of 101 MDS patients and 88 age-matched healthy volunteers, the interest of ten CPD: MN-LALS-NNRBC, MN-LMALS-NNRBC, MN-UMALS-NNRBC, SD-AL2-MO, SD-AL2-NE, MN-MALS-NE, SD-MALS-NE, SD-UMALS-NE, SD-V-MO and SD-V-NE. These parameters were better predictors of MDS than RBC-derived parameters or CPD and were included in an MDS likelihood score (MDS-LS). With a threshold set to zero, the sensitivity and specificity of the MDS-LS were 100% and 80.7% in this cohort. Hwang et al. studied, on a cohort of 64 MDS, 162 non-clonal cytopenia and 119 healthy controls, the CPD derived from leukocytes on an Alinity-hq [18]. Many neutrophil parameters showed significant differences between the MDS and other cytopenia groups, including Neu-ALL-M, Neu-DSS-M, Neu-IAS0-S, Neu-IAS2-S, Neu-IAS3-M, Neu-IAS3-S, Neu-PSS-DK and Neu-PSS-M [18] and so did monocyte and lymphocyte parameters. The interest of lymphocyte-CPD was also recently reported by Di Luise et al. [9] using Sysmex-XN in the differential diagnosis of MDS versus vitamin deficiency anemia. They identified on a cohort of 19 MDSs, 57 vitamin deficiency anemia patients and 56 healthy controls, Ne-WX, Ne-WY, Ne-WZ, Ne-Z and Ly-Y as the most significant parameters. For patients with macrocytic anemia, Ly-Y was helpful to distinguish MDSs from vitamin-deficiency anemia. 

### 2.2. Red Blood Cell–Derived CPD

Macrocytic anemia is a hallmark of MDS, but MCV is usually also increased in a large fraction of non-clonal anemias: liver diseases, endocrinological diseases, vitamin deficiencies, etc. In microscopic examinations of RBCs from MDS patients, anisocytosis and poikilocytosis are also frequently observed, which can be measured by distribution parameters such as RBC distribution width (RDW). Beyond distribution parameters, other parameters related to RBCs can be obtained, such as reticulocyte volume or Hb content or concentration. Regarding hemoglobin measurement, most HAs rely on a spectrophotometric quantification of hemoglobin released from lysed RBCs; therefore, this parameter is unlikely to make a significant contribution to MDS diagnosis. On the contrary, CPD relative to RBC hemoglobin content or concentration would be of interest. However, data related to these parameters are scarce in the literature as they are not systematically available, being only obtained when a reticulocyte count is triggered. Several papers have highlighted the contribution of RBC analysis to MDS diagnosis. Using Sysmex-XN, Boutault et al. showed that MCV was significantly increased in MDS patients versus non-clonal cytopenias in uni- and multivariate analyses and included MCV in their MDS-CBC score [11]. Ravalet et al. also showed on DxH 800 that MCV, RDW, NRBC CPD and low hemoglobin density percentage (LHD) were significantly higher in MDS patients compared to healthy volunteers [12] contrary to a previous report from Kim SY et al., which failed to demonstrate a statistical difference between MDS and three non-clonal cytopenia groups [17]. Recently, Hwang et al., using Alinity-hq, demonstrated that MCV and mean corpuscular hemoglobin (MCH) were higher in MDS patients than in non-clonal cytopenias [18]. They also showed that numerous RBC-derived CPD, including the percentage of macrocytic RBC (MAC), the percentage of hypochromic or hyperchromic RBC, the MCV of reticulocytes and hemoglobin distribution width (HDW) were higher in MDS patients compared to non-clonal cytopenias.

### 2.3. Platelet-Derived CPD

Macroplatelets are a morphological hallmark of MDS, and thus, their presence can impact mean platelet volume (MPV) and platelet distribution width (PDW). When derived from the impedance technology, MPV and PDW can be missing from MDS patients’ CBC (up to 30% of cases in our experience). Indeed, analyzers may be unable to report these parameters due to the presence of large platelets exceeding the upper limit of the normal range of platelet size resulting in platelet impedance histograms that fail to return to the baseline at 20 fL. Ibarra et al. evaluated the IPF%, on a cohort of 37 MDS patients using a Sysmex-XN [19]. They showed that IPF% was correlated to dysplastic thrombopoiesis and that patients with a high IPF% had a trend to a lower overall survival. In link with this observation, Chen et al. recently identified the platelet-large ratio (P-LCR), the percentage of platelets larger than 12 fL, as a prognostic factor in MDS in uni- and multivariate analyses. A low P-LCR fraction was associated with a decreased overall survival in a cohort of 122 MDS patients [20]. Ravalet et al. identified PDW on the DxH 800 as a parameter predictive of MDSs [12]. Hwang et al. also confirmed the interest of PDW using the Alinity-hq [18] showing that plateletcrit was decreased and PDW increased in MDS patients compared to non-clonal-cytopenias. 

## 3. Discussion and Concluding Remarks

**Figure 4:**
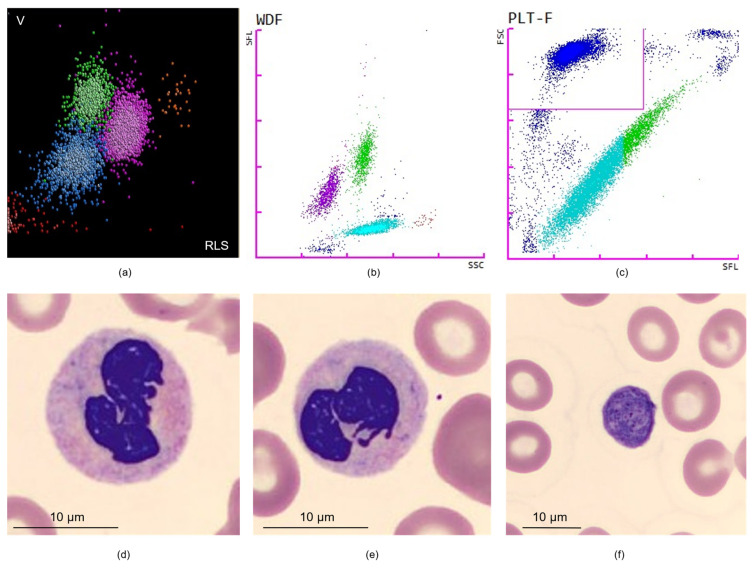
Illustration of typical MDS cases on DxH 800 and Sysmex-XN. (**a**) Leukocyte differential plot from DxH 800, showing decreased light scattering in a patient with myelodysplastic syndrome with excess blasts (MDS-EB-1). LMALS-NE-M, UMALS-NE-M and MALS-NE-M were decreased (128, 127 and 131, respectively). (**b**) Leukocyte differential plot from Sysmex-XN showing an increased Ne-WX (427) in this patient with normal neutrophil count (3.1 × 10^9^/L). This patient had normocytic anemia (Hb 9.7 g/dL, MCV 90 fL) and thrombocytopenia (platelet count: 107 × 10^9^/L), the MDS-CBC score was highly suggestive of MDS (0.755). This patient was diagnosed with MDS with multilineage dysplasia. (**c**) Fluorescence platelet count scatterplot from Sysmex-XN showing increased IPF% (19%) in this patient with macroplatelets on blood smear. (**d**–**f**) Blood film from patient illustrated in (**b**,**c**) showing hypogranular neutrophils (**d**,**e**, magnification ×100) and a macroplatelet (**f**, magnification ×50).

Over the past decade, HAs have undergone rapid development due to technological progress. They show better performances, and the number of parameters reported has increased, as an example, most HAs routinely quantify immature granulocytes and NRBCs. Other new parameters such as CPD are now also reported along with the CBCs. CPD provide quantitative information on the morphological and functional characteristics not only of leukocytes but also of RBCs and platelets. In Figure 4, we have illustrated MDS cases on DxH 800 and Sysmex-XN. As described in Figure 4a,b, a typical abnormality observed in MDS patients is the decreased light scattering of neutrophils. Dysplasia can also be suggested in other lineages by increased MCV (data not shown) or increased IPF% as illustrated in Figure 4c. Microscopic examination of blood smear will then confirm the presence of hypogranular neutrophils (Figure 4d–e) and macroplatelets (Figure 4f). Here, we overviewed technologies from the three main manufacturers, but other technologies exist, such as those developed by Siemens and Mindray, which have not been discussed here. CPD can offer valuable information on the state of activation of the cell and on its functional activity and could be of great interest to follow the efficacy of a treatment. They are parameters generated during CBCs without additional sample needs, available 24/7. In the setting of MDS differential diagnoses, where the purpose is to detect dysplasia, CPD are objective parameters, useful to guide slide review regardless of the laboratory’s slide review criteria. We consider that microscopic reviewing of each CBC is no longer possible due to induced cost (technical and medical time) and the resulting delay in making the results available to the physician. Laboratory specialists can follow international or national guidelines and/or IPSS criteria to guide slide review. Using the IPSS thresholds, patients with one or more cytopenias account for 23% of CBCs in our own experience. MDS diagnosis can be made in patients with very mild CBC abnormalities, for example increased MCV with normal WBC count and differential. As discussed above, HAs are very performant to detect neutrophil dysplasia and it seems appropriate to base our diagnostic work-up not only on simple cell counts but also on morphological information obtained thanks to CPD. 

As reported previously, many authors have studied the contribution of CPD, both individually and also for the development of scores predicting MDSs with outstanding performances allowing faster and cost-effective diagnosis. Given the global implementation of the new generation of HAs, these scores including both cell counts and morphological information obtained thanks to CPD are easily implementable to the routine diagnostic work-up without additional cost for the laboratory. However, the CPD remain raw values, which largely depend on the technology of HAs. The lack of standardization and transferability of each parameter may be a limitation to their widespread use, as well as the difficulty for the laboratory to control or even detect interferences on these data. For usually rare populations such as NRBCs, the minimal quantity of cells allowing the interpretation of CPD should be defined. Reference ranges of normal controls were evaluated in different studies and could be helpful in interpreting these innovative CPD [12,18,21]. The diagnosis of MDS remains challenging due to numerous other non-neoplastic causes of cytopenias. The new generation of HAs provide a reliable set of objective data on each lineage (leukocytes, RBC and platelets). Mining the full potential of CPD using automated scores would benefit from being implemented in routine practice to better perform early MDS diagnosis in a cost-effective way. 

## Data Availability

Not applicable.
